# Supplementary material for: Translation and Validation of the Volunteer Functions Inventory (VFI) among the General Dutch Older Population
Source: Int J Environ Res Public Health. 2019 Aug 26;16(17):3106. doi: 10.3390/ijerph16173106 (PMC6747180; doi:10.3390/ijerph16173106)
Supplement: Supplementary file 1 [file ijerph-16-03106-s001.zip › Table S2.docx]

**Table S2: Sensitivity Analysis comparing CFA factor loadings from ML and ADF estimation**

| **VFI-V scale and items** | **ML** | | **ADF** | |
| --- | --- | --- | --- | --- |
|  | **Coefficient** | **SE** | **Coefficient** | **SE** |
| **1. Understanding**  12. I can learn more about the cause for which I am working  14. Volunteering allows me to gain a new perspective on things  18. Volunteering lets me learn things through direct, hands on experience 25. I can learn how to deal with a variety of people  30. I can explore my own strengths | 1 (constrained)  1.23  1.20  1.14  1.25 | 0.03  0.03  0.03  0.03 | 1 (constrained)  1.28  1.20  1.17  1.22 | 0.03  0.03  0.03  0.03 |
| **2. Career** 1. Volunteering can help me to get my foot in the door at a place where I would like to work 10. I can make new contacts that might help my business or career 15. Volunteering allows me to explore different career options  21. Volunteering will help me to succeed in my chosen profession  28. Volunteering experience will look good on my résumé | 1 (constrained)  1.40  1.34  1.25  1.35 | 0.04  0.03  0.03  0.04 | 1 (constrained)  1.84  1.65  1.65  2.06 | 0.18  0.15  0.17  0.21 |
| **3. Values** 3. I am concerned about those less fortunate than myself  8. I am genuinely concerned about the particular group I am serving 16. I feel compassion toward people in need 19. I feel it is important to help others | 1 (constrained)  0.72  1.08  0.92 | 0.02  0.03  0.03 | 1 (constrained)  0.68  1.00  0.85 | 0.02  0.02  0.02 |
| **4. Protective**  7. No matter how bad I've been feeling, volunteering helps me to forget about it  9. By volunteering I feel less lonely 20. Volunteering helps me work through my own personal problems 24. Volunteering is a good escape from my own troubles | 1 (constrained)  0.96  1.07  0.98 | 0.03  0.03  0.03 | 1 (constrained)  0.81  0.62  0.47 | 0.03  0.03  0.03 |
| **5. Social**  2. My friends volunteer  4. People I'm close to want me to volunteer  6. People I know share an interest in community service  17. Others with whom I am close place a high value on community service  23.Volunteering is an important activity to the people I know best | 1 (constrained)  0.74  1.42  1.43  1.37 | 0.03  0.04  0.05  0.05 | 1 (constrained)  0.64  1.80  2.05  1.61 | 0.03  0.06  0.09  0.06 |
| **6. Enhancement**  5. Volunteering makes me feel important  13.Volunteering increases my self-esteem  26. Volunteering makes me feel needed  27. Volunteering makes me feel better about myself | 1 (constrained)  1.26  1.24  1.28 | 0.03  0.03  0.03 | 1 (constrained)  1.27  1.29  1.22 | 0.02  0.03  0.03 |
