# Supplementary material for: Translation and Validation of the Volunteer Functions Inventory (VFI) among the General Dutch Older Population
Source: Int J Environ Res Public Health. 2019 Aug 26;16(17):3106. doi: 10.3390/ijerph16173106 (PMC6747180; doi:10.3390/ijerph16173106)
Supplement: Supplementary file 1 [file ijerph-16-03106-s001.zip › Table S1.docx]

**Table S1: Dutch translation of the VFI**

Hieronder staan 27 mogelijke redenen voor het doen van vrijwilligerswerk. Wilt u steeds aangeven in hoeverre elk van de genoemde redenen op u van toepassing is?

1. Vrijwilligerswerk kan me helpen een voet tussen de deur te krijgen op een plek waar ik zou willen werken.
2. Mijn vrienden doen vrijwilligerswerk.
3. Ik ben betrokken bij mensen die het minder goed getroffen hebben dan ikzelf.
4. De mensen die dichtbij me staan willen dat ik vrijwilligerswerk doe.
5. Door het doen van vrijwilligerswerk voel ik me belangrijk.
6. Mijn kennissen zijn geïnteresseerd in het leveren van een bijdrage aan de samenleving.
7. Hoe slecht ik me ook voel, vrijwilligerswerk helpt me het te vergeten.
8. Ik ben oprecht betrokken bij de groep die ik help.
9. Door vrijwilligerswerk te doen voel ik me minder eenzaam.
10. Ik kan nieuwe contacten opdoen die mogelijk van pas komen voor mijn bedrijf of carrière.

** (Door vrijwilligerswerk voel ik me er minder schuldig over dat ik het beter getroffen heb dan anderen.)^[[1]](#footnote-1)^*

1. Ik kan meer te weten komen over het doel waarvoor ik me inzet.
2. Vrijwilligerswerk verhoogt mijn gevoel van eigenwaarde.
3. Vrijwilligerswerk stelt me in staat dingen in een nieuw perspectief te zien.
4. Vrijwilligerswerk stelt me in staat verschillende carrièremogelijkheden te onderzoeken.
5. Ik leef mee met mensen die hulp nodig hebben.
6. Mensen die dichtbij me staan hechten veel waarde aan het leveren van een bijdrage aan de samenleving.
7. Door vrijwilligerswerk kan ik dingen leren door directe, praktische ervaring op te doen.
8. Ik vind het belangrijk anderen te helpen.
9. Vrijwilligerswerk helpt me mijn eigen problemen te verwerken.
10. Vrijwilligerswerk zal me helpen succesvol te zijn in mijn beroep.

** (Ik kan iets doen voor een doel dat belangrijk voor me is.)^[[2]](#footnote-2)^*

1. Vrijwilligerswerk is een belangrijke bezigheid voor de mensen die ik het beste ken.
2. Vrijwilligerswerk is een goede afleiding van mijn eigen problemen.
3. Ik kan leren omgaan met verschillende soorten mensen.
4. Vrijwilligerswerk geeft me het gevoel dat ik nodig ben.
5. Vrijwilligerswerk geeft me een beter gevoel over mezelf.
6. Ervaring met vrijwilligerswerk staat goed op mijn cv.

** (Vrijwilligerswerk is een manier om nieuwe vrienden te maken.)^[[3]](#footnote-3)^*

1. Ik kan mijn eigen sterke punten verkennen.

1 = helemaal niet van toepassing / 7 = heel erg van toepassing.

1. This item has been deleted in the validation process and is therefore not part of the Dutch version of the VFI. Originally, this item is stated as Item K (‘Doing volunteer work relieves me of some of the guilt over being more fortunate than others’ [↑](#footnote-ref-1)
2. This item has been deleted in the validation process and is therefore not part of the Dutch version of the VFI. Originally, this item is stated as item V (‘I can do something for a cause that is important to me’) [↑](#footnote-ref-2)
3. This item has been deleted in the validation process and is therefore not part of the Dutch version of the VFI. Originally, this item is stated as item CC (‘Volunteering is a way to make new friends’) [↑](#footnote-ref-3)
